# A New Case Manager for Diabetic Patients: A Pilot Observational Study of the Role of Community Pharmacists and Pharmacy Services in the Case Management of Diabetic Patients

**DOI:** 10.3390/pharmacy8040193

**Published:** 2020-10-19

**Authors:** Raffaele La Regina, Dario Pandolfi, Nicola Stabile, Lucio Beloni, Fulvio Glisenti, Paola Griggio, Micaela La Regina, Giuseppe La Regina

**Affiliations:** 1 Community Pharmacy, Farmacia La Regina, 84030 San Rufo (SA), Italy; 2 Community Pharmacy, Farmacia Pandolfi, 84016 Pagani (SA), Italy; info@farmaciapandolfi.it; 3 Community Pharmacy, Farmacia Stabile, 80125 Napoli (NA), Italy; nicola.stabile@gmail.com; 4 Research Department, Biochemical System International, 52100 Arezzo (AR), Italy; lucio.beloni@biosys.it; 5 Research Department, Health Telematic Network, 25124 Brescia (BS), Italy; fglisenti@e-htn.it; 6 Research Department, Next Sight, 35010 Limena (PD), Italy; paola.griggio@nextsight.info; 7 S.S. Risk Management, ASL 5 La Spezia, 19121 La Spezia (SP), Italy; micaela.laregina@asl5.liguria.it; 8 Department of Drug Chemistry and Technologies, Sapienza University of Rome, 00185 Roma, Italy; giuseppe.laregina@uniroma1.it

**Keywords:** diabetes, pharmacist, pharmacy services, clinical pathway, case management, interprofessional care, interprofessional cooperation, telemedicine, community pharmacy, health-system transformation

## Abstract

The adherence of type 2 diabetes mellitus (DM2) patients with an individual care plan (ICP) is often not satisfactory, nor does it allow for a significant improvement in outcome, because of poor accessibility to services, poor integration of pathway articulations, poor reconciliation with the patient’s life, or the lack of a constant reference person. The purpose of this study was to evaluate the contribution of community pharmacists and pharmacy services in improving adherence with periodic controls in DM2. The study was conducted at a rural pharmacy. A sample of 40 patients was calculated with respect to a historical cohort and subsequently enrolled. Clinical and personal data were collected in an electronic case report form. Pharmacists acting as a case manager followed patients carrying out their ICP developed by an attending physician. Some of the activities foreseen by the ICP, such as electrocardiogram, fundus examination, and self-analysis of blood and urine, were carried out directly in the pharmacy by the pharmacist through the use of telemedicine services and point of care units. Activities that could not be performed in the pharmacy were booked by the pharmacist at the accredited units. Examination results were electronically reported by the pharmacist to the attending physician. The primary endpoint was the variation in patient adherence with the ICP compared to a historical cohort. Secondary endpoints were variation in waiting time for the examinations, mean percentage change in glycated hemoglobin (HbA1c) and low-density lipoprotein (LDL) cholesterol levels and blood pressure, impact on healthcare-related costs, and perceived quality of care. Adherence to the ICP significantly increased. Waiting times were reduced and clinical outcomes improved with conceivable effects on costs. Patients appreciated the easier access to services. Community pharmacists and pharmacy services represent ideal actors and context that, integrated in the care network, can really favor ICP adherence and obtain daily morbidity reduction and cost savings through proper disease control and an early diagnosis of complications.

## 1. Introduction

Type 2 diabetes (DM2) is the most common form of diabetes, representing almost 90% of all cases of the disease. According to the International Diabetes Federation [1], today, there are globally about 463 million people suffering from diabetes mellitus, and this number will reach 700 million by 2045. Globally, diabetes mellitus is one of the main determinants of morbidity, mortality, and healthcare-related costs due to its numerous and serious complications.

Recent pharmacoeconomic studies published by Sole24ore [2] showed that half of the direct diabetes-related costs are due to hospitalizations, and about one-half are due to drugs, especially those used for the treatment of complications. Thus, better disease control and the tight prevention of any complications through careful and close monitoring could represent an effective tool to reduce any negative clinical and economic impact at the individual and system level. For this reason, in many countries, clinical care pathways (CCPs) and subsequent individual care plans (ICPs) have been developed. However, patient adherence with an ICP is often not satisfactory, nor does it allow for a significant improvement in outcome.

In Italy, an interesting research project coordinated by the Health and Social Agency of the Emilia Romagna region [3] explored facilitating factors and barriers to the adherence of diabetic patients to CCPs. Among the obstacles, they listed:

- Poor accessibility to controls,
- Poor integration of the different pathway articulations,
- Poor reconciliation with the patient’s life and work rhythms.

Among the facilitating factors, they highlighted: the presence of a constant reference person with relational and listening skills who is able to motivate patients and give answers on several fronts.

Indeed, most management models present in Italy involve services provided on an outpatient basis at the hospital and/or health-district level; thus, they are accessible only in the daytime and not on weekends, and they are subject to shift rotations.

On such a basis, studying new management models could be a way of overcoming the critical organizational issues highlighted above and of guaranteeing the ability to meet needs expressed by patients who are also seeking new figures, such as community pharmacists and new structures, such as innovative telemedicine technologies, to support traditional ones. Indeed, there is evidence of how case management, combined with telemedicine services, can significantly improve clinical outcomes [4,5,6].

In Italy, since 2009, community pharmacists have been given the opportunity to take part in the management of patients with chronic diseases, and to provide telemedicine services, urinalysis, and blood self-analysis, creating expanded pharmacy services. However, to date, no projects have been carried out demonstrating the real potential of this model. Community pharmacies, in fact, could be the perfect partner for the management of chronic patients thanks to their convenience, daily hourly availability, and provided services.

The main objective of this pilot study was to explore the unexpressed potential of pharmacy services and, above all, of community pharmacists in the management of chronic diseases, i.e., type 2 diabetes, alongside general and specialist medical practitioners in order to sustainably improve the current quality of diabetes care.

## 2. Materials and Methods 

### 2.1. Design

This is an observational, prospective, monocentric, non-controlled, nonprofit (without any economic financing) pilot study, lasting 12 months (November 2018–October 2019). 

The primary endpoint of the study was variation in patient adherence with the ICP. Enrolled patients were followed by community pharmacists acting as case managers in the setting of pharmacy services; they were compared to a historical cohort that was traditionally managed. The historical cohort was that from the previously mentioned study conducted by the Health and Social Agency of the Emilia Romagna region, in which the degree of adherence to the ICP was 0.6%.

The variation in patient adherence to the ICP was calculated using the following equation: patients who performed the scheduled checks at 3, 6, and 12 months/total patients enrolled × 100.

Secondary endpoints of the study were as follows:

- Variation in waiting time for obtaining an appointment for the examinations included in the ICP;
- Mean percentage change in glycated hemoglobin (HbA1c) and low-density lipoprotein (LDL) cholesterol levels, and blood pressure;
- Reduction in healthcare-related costs;
- Improvement in perceived quality of care.

### 2.2. Sample and Setting

Sample size was calculated as a function of adherence to all CCP controls measured in a historical cohort of the Emilia Romagna region (0.6%) with the aim of raising the percentage of adherence to 18%, with an error of α = 0.05 and a statistical power (error 1-β) of 95%. The calculated sample of 20 patients was doubled to 40 to remedy any abandonment or withdrawal of consent during the study. All participants gave their informed consent for inclusion before they participated in the study. The inclusion criteria were as follows: (1) age > 18 years old; (2) diagnosis of type 2 diabetes mellitus; and (3) ability to express informed consent.

This study was conducted at a rural community pharmacy operating under an agreement with the Italian National Health System. Eight general practitioners and two specialists in diabetes care from the local health unit were also involved.

### 2.3. Intervention

The study design included the following phases:

Phase 1—patients screening: Patients with inclusion characteristics were identified and enrolled.

Phase 2—enrollment confirmation and definition of ICP: Enrollment was reported to one’s attending physician and a request was made to fill in the ICP. Each attending physician provided for the insertion of personal data and clinical history of their patients in an electronic case report form (CRF), as reported in the next paragraph.

Phase 3—pharmacist case management activities and data collection: a community pharmacist acting as case manager followed any patient carrying out the examinations required by the ICP, drawn up by their attending physician according to the regional CCP. Electrocardiogram, fundus examination and self-analysis of blood and urine (glycated hemoglobin, lipids, and microalbuminuria) were carried out directly in the pharmacy by the pharmacist through the use of telemedicine services and point of care units. Activities that could not be performed in the pharmacy were booked by the pharmacist at the accredited units. Examination results were electronically reported by the pharmacist to the attending physician as soon as they were available.

Phase 4—measurement of perceived quality and analysis of results: An anonymous questionnaire was administered to patients to measure the perceived quality. Subsequently, after 12 months, the collected data was analyzed.

### 2.4. Data Collection

For any patient, the following clinical and personal data were collected in an electronic case report form (CRF), shared with attending physicians:

- General information (gender, age, etc.);
- Clinical history;
- Vital signs and blood analysis on entry;
- List of exams and procedures required by ICP;
- Results of examinations performed according to ICP;
- Answers to the perceived quality-assessment questionnaire.

### 2.5. Data Analysis

Biohumoral and clinical parameters, and the adherence to prescribed examinations were assessed every 3–6 months depending on the ICP. Variation in waiting time for due examinations was calculated by constantly monitoring the waiting times released by the booking centers of the local healthcare unit during the study period. Economic evaluation was performed by comparing the costs of the study model (pharmacy-based) with those of the traditional one (hospital-based), including savings derived from the estimated reduction in complications and the increase in drug expenses to achieve better disease control. Perceived quality was evaluated using a short anonymous questionnaire administered to all enlisted subjects at the end of the study, aimed at verifying if the model overcame the critical issues that emerged from the research carried out by the Health and Social Agency of the Emilia Romagna region.

All categorical variables measured in this study are reported as absolute numbers and percentages. Continuous variables are reported as mean and standard deviation or median and interquartile range (IQR), depending on the normal or non-normal distribution of the data. Differences were analyzed using chi-squared tests, t-tests for unpaired data, or Mann–Whitney U tests, depending on the nature of the variable. Any difference with *p* < 0.05 was considered statistically significant.

### 2.6. Ethical Considerations 

The study was conducted in accordance with the Declaration of Helsinki, and the protocol was approved by the Ethics Committee Campania Sud (authorization no. 169; 31 October 2018) and registered on clinicaltrials.gov (NCT03752567).

## 3. Results

### 3.1. Patient Characteristics

Forty patients (50% female; mean age, 64.5 ± 9.53 years) were enrolled. The study population’s descriptive data are summarized in Table 1.

### 3.2. Primary Endpoint

Adherence of the enrolled patients to the ICP (primary study endpoint) was 98% in the first quarter and 100% in the remaining three quarters (*p* < 0.00001) compared to the historical reference cohort.

### 3.3. Secondary Endpoints

The use of technologies based on specialist teleconsultation and remote reporting allowed for performing diagnostic tests approximately 120 days in advance (ECG, 117.2 days (SD 57.63); fundus oculi, 120 days (SD 50.2)) in comparison with the local health unit.

**Figure 1:**
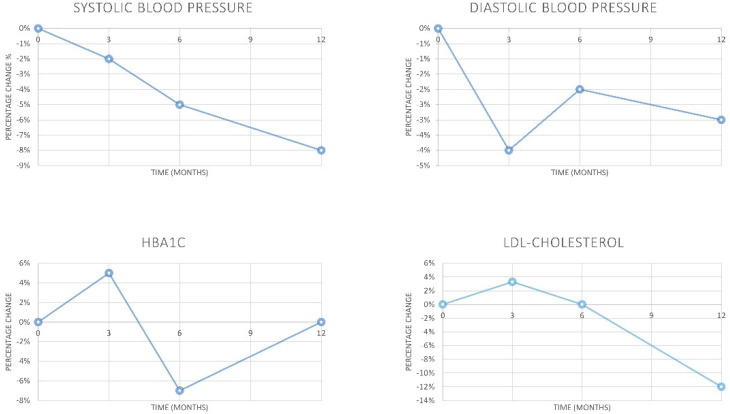
Percentage change over time in monitored biochemical parameters.

The mean percentage change in biological and clinical parameters between the start and the end of the study (0–12 months) was −4% for HbA1c (SD ± 5), −10% for LDL cholesterol (SD ± 7), −13% for systolic blood pressure (BPSys) (SD ± 4), and −9% for diastolic blood pressure (BP Dia) ((SD ± 2), with maximal reductions of −7% for HbA1c between the first and second quarters (3–6 months), and −17% for LDL cholesterol in the first semester (0–6 months). Further details are shown in Figure 1.

Assuming that these percentage changes would result in a 10% reduction in complications and an increase in drug expenses, as estimated by Marcellusi et al. [7], they would result in savings of EUR 10,000/year on average for every 40 patients (see details in Table 2). 

The multiple-choice questionnaire investigating perceived quality revealed that better patient information and easier accessibility to services, as reported by 80% of the participants (see details in Table 3), were the strengths of our model.

## 4. Discussion

Patients with type 2 diabetes mellitus represent a particular kind of chronic patient who has little awareness of the potential evolution of the disease due to poor symptomatology, scarce information (mainly in the initial stages), and the unavailability of an easily accessible and motivational reference person.

According to data on adherence available in Italy, to successfully manage such patients, it is necessary to completely rearrange the care model to ensure a case manager who can constantly follow the patient, resolve any doubt, and guide them with respect to their ICP. Easy access to diagnostic activities to better control the disease and prevent complications is also mandatory. Patients need easy, streamlined models of care, able to satisfy disease-related needs at any time of the day and under any circumstances. In fact, current management models do not fit well with the hectic lifestyles of our time.

The management model, i.e., the object of our study, showed strong impact on adherence to the ICP; 98% of enrolled patients performed all examinations included in the ICP within the first quarter, while 100% performed all examinations in the remaining three quarters.

Improvements in clinical and biological parameters, as shown in Figure 1, were highest when the interaction between general practitioner and pharmacist was stronger, as, in Italy, pharmacists are not allowed to prescribe drugs or refer patients directly to medical specialists; this role applies only to general practitioners (GPs). Patients assigned to more collaborative GPs showed improved disease control within the second quarter, which was maintained until the end of the study, while the trend of decline in others who continued to worsen was determined after the second quarter. Furthermore, the vacancy of the position of director at the local diabetes center negatively impacted clinical endpoints after the second quarter.

As highlighted by the United Kingdom Prospective Diabetes Study Group [8], a reduction in glycated hemoglobin values of 1%, resulting from the intensive control of blood glucose values, is directly connected to a reduction in the relative risk of microvascular complications (−37%), death from diabetes (−21%), and myocardial infarction (−14%). Thus, any management model, such as this one, which is able to significantly reduce the incidence of complications, can have strong impact on patient morbidity, mortality, quality of life, and healthcare-related costs.

Pharmacist activity, historically associated with final control over medical prescriptions, has evolved over time. They have developed new skills, aimed at creating so-called pharmacy services, representing a reality where pharmacists become actors in a new version of medicine. 

Community pharmacists and pharmacy services represent yet unexploited potential in the management of chronic patients, particularly diabetics. Taking on the role of case manager for patients suffering from type 2 diabetes mellitus, pharmacists could achieve tangible improvements in terms of adherence to controls and therapies, as well as in terms of clinical and economic outcomes, thanks to the convenient diffusion of pharmacies, their hourly availability, the skills possessed by pharmacists, and the innovative services they can offer, such as telemedicine services. In this scenario, pharmacists do not replace any other actors of the current multidisciplinary chronic care team; instead, they integrate into it without generating excessive additional costs, i.e., new hires, since, in countries like Italy, community pharmacists already act in agreement with the national health service. Thus, the integration of community pharmacists in the case management of type 2 diabetes could represent the sustainable key for the long-awaited dehospitalization of chronic patients, which has been talked about for a long time without finding concrete and relatively inexpensive solutions.

Evidence regarding the involvement of pharmacists in the chronic management of type 2 diabetic patients is limited and heterogeneous, albeit positive. In general, in studies where pharmacists were involved in the diabetes care team, a significant improvement in therapeutic outcomes, and a reduction in hospitalizations and consumption of resources were observed [9,10,11,12,13,14,15,16]. These effects are more evident when prescription abilities are ascribed to the pharmacist (an activity not allowed by Italian law). With regard to telemedicine in pharmacy, most experiences reported in the literature refer to the telemonitoring of blood glucose levels [17] in rural and isolated areas [18,19,20]. However, point-of-care (PoC) equipment and remote-reporting systems (electrocardiogram, ocular fundus, etc.) have reached a maturity that allows for them to be used without reserve in the clinical management of patients.

The present study investigated a completely new scenario in which community pharmacists worked as case managers of type 2 diabetes patients, with the pharmacy becoming a sort of smart clinic. The study was carried out in a rural setting; however, given the ubiquitous presence of chronic patients and the congestion of healthcare units, its potential can also be appreciated in urban areas. A workload reduction in healthcare units, achieved through the integration of pharmacy services in the CCP, would allow for more time for the care of patients who require further investigation rather than screening for complications, as well as a reduction in costs related to personnel and infrastructure. Furthermore, the opportunity to have examinations done at any time of day certainly allows for an improvement in the adherence of patients to their ICP, the rapid identification of complications, and the early start of appropriate treatment, as clearly shown in our assessment of perceived quality.

Finally, this study can be a model for other countries for several reasons:

1. The services of telemedicine and self-analysis of blood and urine through the use of point of care units are now spread fairly evenly in the various countries of the world within the network of pharmacies.

2. The management of type 2 diabetes mellitus appears to be almost similar in different countries in relation to the clinical and instrumental tests that the patient must carry out for the control of the disease and the prevention of complications.

The monometric nature of our study and the small number of enrolled patients, even if statistically adequate, represent the biggest limitation of our study, the results of which need to be confirmed on a larger scale. Furthermore, the high prevalence of type 2 diabetes and the limited healthcare offered in Campania (study area) do not allow for the immediate generalizability of the study results. A further limitation is represented by the country-specific role of pharmacists in the national health system (employee or agreement), as, in Italy, pharmacists neither have the ability to administer prescriptions nor refer patients directly to medical specialists.

Some model limitations are:

The high cost of equipment for the provision of telemedicine and self-analysis services, which smaller pharmacies with fewer users may find difficult to sustain.

The lack, at present, of an adequate training course for the management of patients with type 2 diabetes.

The lack of integration of community pharmacies into chronic disease management programs.

## 5. Conclusions

To the best of our knowledge, this is the first study conducted in Italy testing the putative role of pharmacists as case managers in the setting of diabetes. Community pharmacists and pharmacy services represent ideal actors and context that, integrated in the clinical pathway, can really favor ICP adherence, and obtain a daily morbidity reduction and cost savings through proper disease control and an early diagnosis of complications. As demonstrated by this pilot study, having a constant reference figure in one’s daily life, and being able to easily access diagnostic activities are key factors for adherence to the ICP of type 2 diabetic patients.

The adoption of a model such as the one under study could have the following implications in:

Education: a reorganization of the university courses of study of the faculty of pharmacy in order to provide pharmacists with the appropriate skills to become the case manager of the patient suffering from type 2 diabetes mellitus.

Research: the possibility of having an increasing number of clinical cases available as a consequence of the greater adherence to check-ups, and consequently being able to use the data collected for the improvement of clinical paths, technologies, and drug therapies.

Policy Making: the possibility of creating more streamlined care paths thanks to increased accessibility to services which, in the long term, would allow the generation of economic savings useful for the continuous improvement of the National Health Service.

## Figures and Tables

**Table 1:** Patient characteristics at baseline (n = 40).

Characteristics	*n* (%)
Demographic data	
Sex, female	20 (50)
Age, mean (SD)	64.5 (9.53)
Health status	
Comorbidities, median (SD) (HIV infection, Dementia, Neoplasm, Liver disease, Kidney disease, Peripheral vascular disease, Respiratory disease, Cardiovascular disease, Disease of the nervous system, Non-neoplastic hematological diseases, Rheumatic diseases, Gastro-entero-pancreatic diseases, other disease)	2.0 (1.8)
Stage of diabetic disease	
Reduced glucose tolerance	6 (15)
Altered fasting blood sugar	2 (5)
Type 2 diabetes without organ complications	15 (38)
Type 2 diabetes with chronic complications	17 (43)

**Table 2:** Economic impact of pharmacist-based model. Change in costs as a function of continuous monitoring of glycated hemoglobin (Hba1c), microalbuminuria (M-ALB), low-density lipoprotein (LDL) cholesterol (COL.), and blood pressure (BP).

Base Case	Hba1c	Hba1c + M-ALB	Hba1c + M-ALB + COL.	Hba1c + M-ALB + COL. + BP
EUR 92,770.80	EUR 88,867.08	EUR 84,159.65	EUR 81,404.08	EUR 83,011.5

**Table 3:** Results of perceived quality assessment.

Questions	Answers		
	%	*N*	Choice
Are you aware of the investigations that need to be carried out every year to control disease and chronic complications?	93	37	Yes
	7	3	No
Who informed you about the therapeutic diagnostic path to follow?	30	12	General practitioner
	90	36	Community pharmacist
	10	4	Medical specialist
In the past two years, did you carry out all the planned checks?	78	31	Yes
	22	9	No
From your experience with this study, do you believe that pharmacists can be the most suitable figure to follow you during your treatment and control of the disease?	100	40	Yes
	0	0	No
What did you like most about the pharmacists’ activities during the study?	23	9	Self-analysis
	28	11	Telemedicine services
	13	5	Reminder services
	15	6	Counseling services
	28	11	Constant availability
	28	11	Clarity of provided information
	80	32	Ease of access to services
